# Aflatoxin B_1_ Negatively Regulates Wnt/β-Catenin Signaling Pathway through Activating miR-33a

**DOI:** 10.1371/journal.pone.0073004

**Published:** 2013-08-27

**Authors:** Yi Fang, Youjun Feng, Tongjin Wu, Swaminath Srinivas, Weiqiang Yang, Jue Fan, Chi Yang, Shihua Wang

**Affiliations:** 1 The Ministry of Education Key Laboratory of Biopesticide and Chemical Biology and the College of Life Sciences, Fujian Agriculture and Forestry University, Fuzhou, P. R. China; 2 Academy of Integrative Medicine, Fujian University of Traditional Chinese Medicine, Fuzhou, P. R. China; 3 Fujian Key Laboratory of Integrative Medicine on Geriatrics, Fujian University of Traditional Chinese Medicine, Fuzhou, P. R. China; 4 Department of Microbiology, University of Illinois at Urbana-Champaign, Champaign, Illinois, United States of America; 5 Department of Biochemistry, University of Illinois at Urbana-Champaign, Champaign, Illinois, United States of America; National Cancer Center, Japan

## Abstract

MicroRNAs are known to play an important role in modulating gene expression in various diseases including cancers and cardiovascular disorders, but only a few of them are associated with the pathology of aflatoxin B_1_ (AFB_1_), a potent mycotoxin. Here, we discovered a novel regulatory network between AFB_1_, miR-33a and β-catenin in human carcinoma cells. The level of miR-33a was up-regulated in hepatocellular carcinoma (HCC) cells treated with AFB_1_, while in the same cells causing the decrease in β-catenin expression when treated at their IC_50_ values. miR-33a, specifically miR-33a-5p, was demonstrated to down-regulate the expression of β-catenin, affect the β-catenin pathway, and inhibit cell growth. Also, by employing a luciferase assay, we found that miR-33a down-regulated β-catenin by directly binding to the 3’-UTR of β-catenin. These results suggested that AFB_1_ might down-regulate β-catenin by up-regulating miR-33a. This understanding opens new lines of thought in the potential role of miR-33a in the clinical therapy of cancer.

## Introduction

Aflatoxins are secondary metabolites produced by *Aspergillus parasiticus* (aflatoxins B_1_, B_2_, G_1_, and G_2_) and *Aspergillus flavus* (aflatoxins B_1_ and B_2_) with aflatoxin B_1_ (AFB_1_) being the most prevalent toxin. These aflatoxins producing by members of *Aspergillus* commonly contaminate food, especially peanuts and corn. In humans, evidence has shown that acute aflatoxicosis could cause vomiting, disease of the liver and heart, pulmonary edema, coma and even death [1,2]. Being one of the most critical hepatocarcinogenic factors in many animal species [3–5], AFB_1_ exposure typically leads to hepatocellular carcinoma (HCC) through prolonged dietary exposure along with other risk factors including the hepatitis B virus (HBV), hepatitis C virus (HCV) or heavy alcohol intake. AFB_1_ is accumulated and metabolized predominantly in the liver, and its toxicity requires cytochromes P450 (CYPs) like CYP1A2, CYP3A4 and CYP2A6 in the liver for its metabolic activation [6–8]. These enzymes usually catalyze AFB_1_ to AFB_1_-8,9-exo-epoxide (exo-AFBO), which is a putative reactive intermediate and carcinogenic epoxide [9]. Exo-AFBO exhibits toxicity by binding to nucleic acids and proteins [10]. Exposure to aﬂatoxin B_1_ leads to accumulation of DNA adducts, *p53* gene mutation in hepatocellular carcinoma [11], and overexpression of β-catenin [12]. In addition to the accumulation of β-catenin, mutations in *CTNNB1*, the gene encoding β-catenin, were reported at a low frequency in HCC in response to high AFB_1_ exposure [12–15]. This suggested that other unidentified biomolecules modulating β-catenin stability may be involved in aflatoxin-associated HCC, and that these molecules might either be miRNAs or the products of mutations of another Wnt/β-catenin signaling components [13].

β-catenin is a subunit of the cadherin-associated protein complex which constitute the adherens junctions. It has also been implicated to be an essential component in the well-known Wnt/β-catenin signaling pathway. β-catenin also plays an important role in cell differentiation, proliferation, apoptosis, metastasis and tumorigenesis [16,17]. In normal cells, β-catenin is always controlled at a proper level by the phophorylation of GSK-3β, while the mutation and accumulation of β-catenin always lead to cancer [18–21]. β-catenin not only regulates the basal expression levels of CYPs, but also controls the magnitude of induction and hepatic localization of the response to xenobiotic inducers [22].

microRNAs (miRNAs) are a class of small noncoding RNA molecules, which are expressed endogenously and around 20~25 nt long [23,24]. miRNAs could negatively regulate gene expression through base-pairing with complementary sequence within messenger RNAs (mRNAs). They usually bind with partial complementarity to the 3’ untranslated region (3’-UTR) of the corresponding target mRNA [25]. These interactions can either cause target mRNA degradation or translational repression which would lead to activation or inhibition of downstream signaling pathways. Some miRNAs are also found to regulate target genes in other ways, via binding to their targets in coding regions [26] or long non-coding RNA [27,28]. miRNAs are involved in various biological processes, including cell growth, proliferation, apoptosis and differentiation [29–34]. Studies focusing on the abnormal expression of miRNAs in human cancers have suggested that the presence of miRNA could have some consequential effect on tumorigenesis [35,36], such as hepatocarcinogenesis [21,37,38].

To date, several human miRNAs have been shown to interact with β-catenin. For example, Xia et al. identified that miR-200a regulates epithelial-mesenchymal transition with β-catenin as its downstream target in nasopharyngeal carcinoma cells [39]. Down-regulated miR-200a in meningiomas could also promote tumor growth by its involvement in the β-catenin signaling pathway [40,41]. The post-transcriptional activity of β-catenin is inhibited by miR-483-3p [26] and bioinformatic analysis shows that miR-125 and miR-214 might be miRNAs that putatively target β-catenin.

So far, very few studies have implicated the function of miRNAs in AFB_1_ induced development of hepatocarcinogenesis. As the dysregulation of miRNAs is always associated with diseases, we believe that some of them should be involved in the pathogenic and carcinogenic mechanism of AFB_1_. In this paper, we discovered for the first time a connection between AFB_1_, β-catenin and miRNA in human carcinomas cells. To predict the miRNAs that target β-catenin, we performed a bioinformatic search for speciﬁc miRNAs that might bind to the β-catenin 3’-UTR. Six of the predicted target miRNAs were overexpressed in cells, and a translational down-regulation of β-catenin levels by miR-33a and miR-340 was observed. Luciferase assay identified that miR-33a controlled β-catenin expression by directly binding to the 3’-UTR. Furthermore, qRT-PCR results showed that miR-33a also down-regulated β-catenin at a post-transcriptional level. It was also found that miR-33a inhibited hepatoma cell colony formation and viability. Meanwhile, it was determined that a down-regulation of β-catenin in cells treated with AFB_1_ was accompanied with the increase of miR-33a levels. Together, these results indicated that miR-33a mediated the toxicity of AFB_1_ by negatively regulating β-catenin activities and functions. These findings may offer an increased understanding of miR-33a regulation, and provide novel clues for the role of miRNAs in the mechanism of carcinogenesis induced by AFB_1_ and ways for prevention of HCC. 

## Results

### Exposure to aﬂatoxin B_1_ leads to β-catenin down-regulation

**Figure 1:**
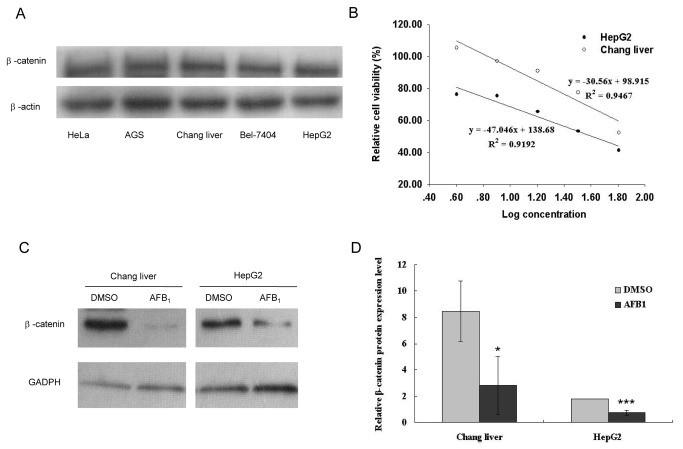
Aﬂatoxin B_1_ exposure down-regulates β-catenin expression in hepatoma carcinoma cell. (A) The protein levels of β-catenin in HeLa, AGS, Chang liver, Bel-7404 and HepG2 cell lines. (B) Relative cell viability of cells treated with AFB_1_. (C) β-catenin protein levels are decrease in Chang liver and HepG2 cells treated with AFB_1_ at their IC_50_ value. (D) Relative levels of β-catenin protein in cells treated with AFB_1_. The β-catenin protein levels were normalized with GADPH in (C). Data is presented as mean ± SD. *P < 0.05, **P < 0.01, ***P <0.001.

There is evidence that β-catenin is always activated in hepatocellular carcinoma. In our study, we first examined the β-catenin protein levels in five kinds of cell lines including Chang liver (liver), HepG2 (liver), Bel-7404 (liver), HeLa (cervix) and AGS (stomach). β-catenin was expressed in all these cell lines, and their expression levels had no significant differences (Figure 1A), indicating that β-catenin is a widely expressed protein in many kinds of cells. As AFB_1_ is predominantly accumulated and metabolized in the liver, Chang liver cells (normal liver cell line) and HepG2 cells (hepatoma cell line) were selected for further experiments. MTT assay was employed for the determination of cell viability of two cells lines. IC_50_ values were calculated from the exponential equations shown in Figure 1B, and IC_50_ values of 40 μg/mL and 77 µg/mL were obtained for Chang liver and HepG2 respectively. Subsequent treatment of the these two cells with AFB_1_ at their IC_50_ value, followed by a western blot showed that β-catenin was down-regulated after AFB_1_ exposure in both cell lines (Figure 1C and 1D).

### Overexpression of miR-33a decreases protein levels of β-catenin

**Figure 2:**
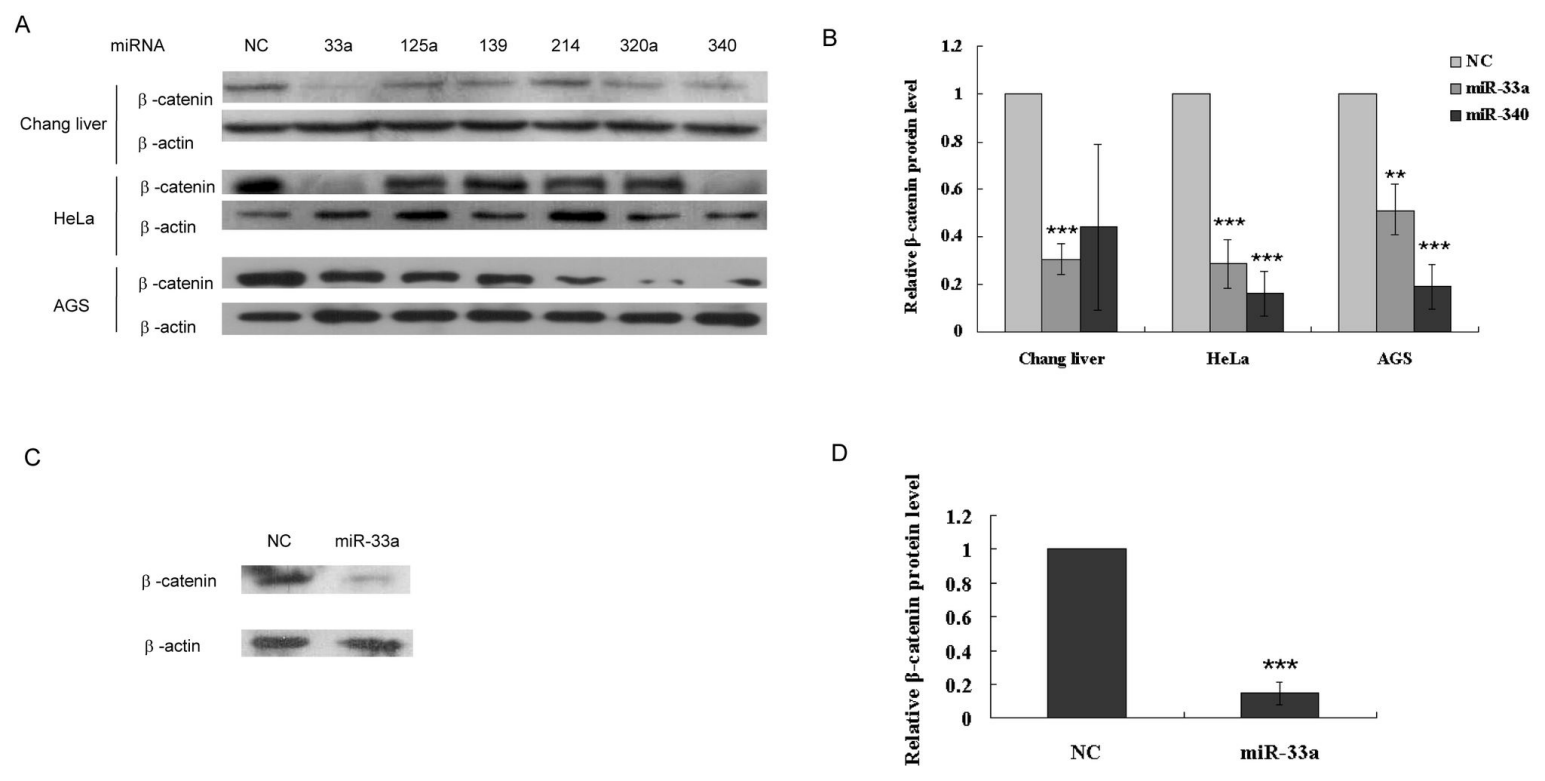
miR-33a negatively regulates β-catenin levels in cells. (A) β-catenin protein levels in Chang liver, HeLa and AGS cells transfected with different miRNA expression vectors for 48 h. (B) Relative levels of the β-catenin protein after transfection for 48 h in Chang liver, HeLa and AGS cells. The β-catenin protein levels were normalized with β-actin in (A). (C) miR-33a down-regulates β-catenin protein levels in HepG2 cells after transfection for 48 h. (D) Relative levels of the β-catenin protein after transfection for 48 h in HepG2 cells. The β-catenin protein levels were normalized with β-actin in (C). Data is presented as mean ± SD. *P < 0.05, **P < 0.01, ***P <0.001.

In order to search for putative miRNAs which directly target the 3'-UTR region of the human β-catenin, online computational algorithms such as PicTar (http://pictar.bio.nyu.edu/), miRNA Viewer (http://cbio.mskcc.org/cgi-bin/mirnaviewer/mirnaviewer.pl) and Targetscan (http://www.targetscan.org/) were employed. miR-320a, miR-33a, miR-139, miR-340, miR-214 and miR-125a were predicted to be the better candidates based on the number of binding sites and the frequencies of prediction by three computational algorithms (Table S1). To validate these six candidates, miRNA over-expression vectors were constructed. Since the miRNA displays the same function regardless of the type of cell line, it might function as part of a fundamental biological pathway. For this consideration, the three different kinds of cell lines, Chang liver, Hela and AGS cells, were chosen for the transfection. After transfection, β-catenin expression was detected by western blot. Result showed that both miR-33a and miR-340 decreased β-catenin protein levels in all three cell lines by 2- to 5-fold (Figure 2A and 2B). Since the effect of miR-340 in Chang liver was not consistent in this study, miR-33a was chosen for further studies. In addition, the expression of β-catenin was also down-regulated by miR-33a treatment in HepG2 cells (Figure 2C and 2D). These results clearly indicated that miR-33a could negatively regulate β-catenin at protein levels.

### miR-33a decreases the mRNA levels of β-catenin and relative genes of β-catenin signaling pathway

**Figure 3:**
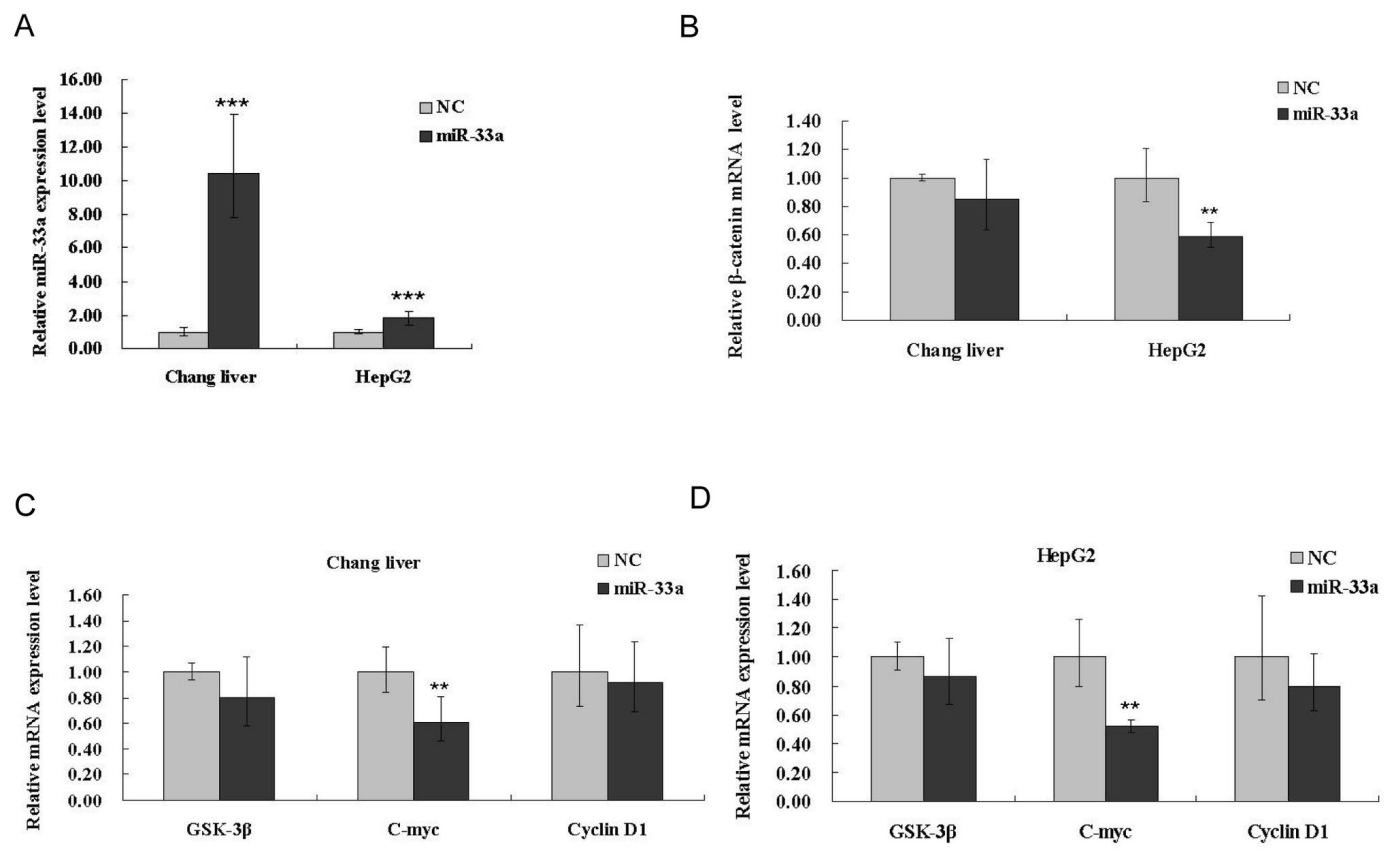
miR-33a decreases the post-transcriptional activity of β-catenin and related genes in the β-catenin signaling pathway. After transfection of pSilence 4.1-miR-33a or pSilence 4.1 vector (NC) into Chang liver and HepG2 cells for 48 h, cells were collected for the extraction of total RNA for real-time PCR. mRNA levels of all genes were normalized to β2-MG (control). (A) Relative expression level of miR-33a was conﬁrmed by way of real-time PCR. (B) Overexpression of miR-33a down-regulates mRNA levels of β-catenin in Chang liver and HepG2 cells. mRNA expression patterns of the related genes in β-catenin signaling pathway were analyzed in Chang liver (C) and HepG2 (D) cells with miR-33a overexpression. Data is presented as mean ± SD. *P < 0.05, **P < 0.01, ***P <0.001.

Since we observed that over-expression of miR-33a could repress protein levels of β-catenin, we decided to examine if miR-33a played a similar role at a post-transcriptional level. For this purpose, the mRNA levels of β-catenin and its downstream target genes were examined by qRT-PCR after transfection of miR-33a over-expression vectors. The expression of miR-33a was verified after overexpression (Figure 3A), which conﬁrmed the successful transfection of miR-33a. In both two liver cell lines, over-expression of miR-33a reduced β-catenin expression at the post-transcriptional level (Figure 3B), consistent with the result of the western blot (Figure 2). These results indicated that miR-33a could induce post-transcriptional down-regulation of β-catenin. Corresponding to the β-catenin gene, two downstream genes of wnt/β-catenin signaling pathway, C-myc and cyclin D1 were also decreased at the post-transcriptional level (Figure 3C and 3D). Besides, the reduction of C-myc was significantly.

### miR-33a-5p is the mature form which down-regulates the β-catenin gene

**Figure 4:**
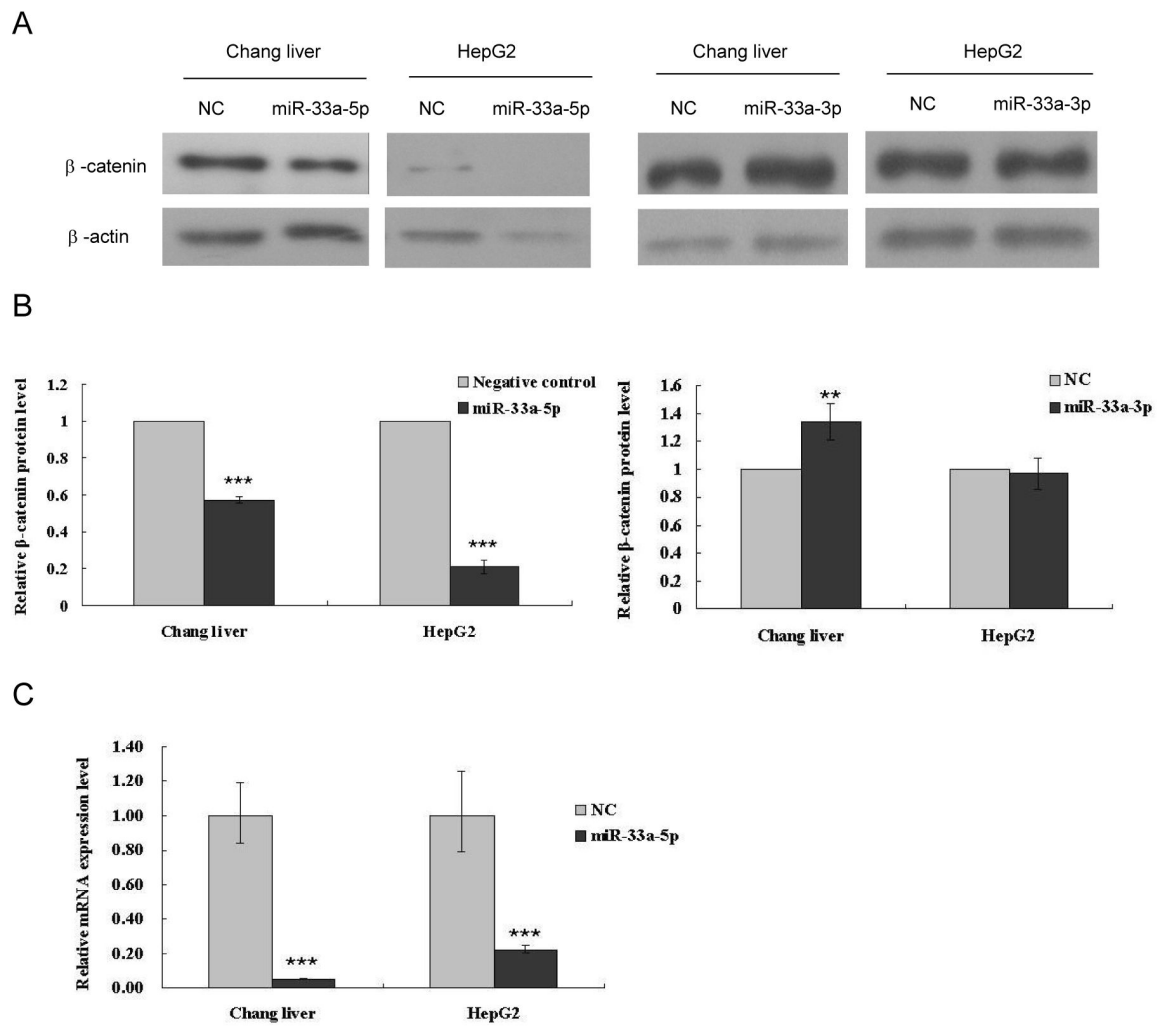
The mature form of miR-33a, miR-33a-5p downregulates the β-catenin gene. (A) β-catenin protein levels in 48 h after miR-33a-5p and miR-33a-3p mimics transfection in Chang liver and HepG2 cells. (B) Relative β-catenin protein levels in 48 h after miR-33a-5p and miR-33a-3p mimics transfection in Chang liver and HepG2 cells. The β-catenin protein levels were normalized relative to β-actin in (A). (C) miR-33a-5p decreases β-catenin mRNA levels in Chang liver and HepG2 cells. Cells were transfected with miR-33a-5p mimics, and mRNA levels of the β-catenin were anaylzed in 48 h. The β-catenin mRNA levels were normalized relative to β2-MG. Data is presented as mean ± SD. *P < 0.05, **P < 0.01, ***P <0.001.

To further examine exact mature form of miR-33a causing the down-regulation of β-catenin, two mature miR-33a (miR-33a-5p and miR-33a-3p) mimics were synthesized, and transfected into Chang liver and HepG2 cells. The expression of β-catenin was then detected by western blot. As shown in Figure 4A and 4B, miR-33a-5p significantly reduced the expression of β-catenin, whereas transfection of miR-33a-3p didn’t show any suppression of β-catenin. Similar results were also observed for the post- transcriptional activity of β-catenin in Chang liver and HepG2 after miR-33a-5p mimics transfection (Figure 4C). These results suggested that miR-33a could suppress β-catenin expression at both post-transcriptional and translational levels, and miR-33a-5p was the final product of pri-miR-33a which could negatively regulate β-catenin.

### miR-33a inhibits HCC cell growth

**Figure 5:**
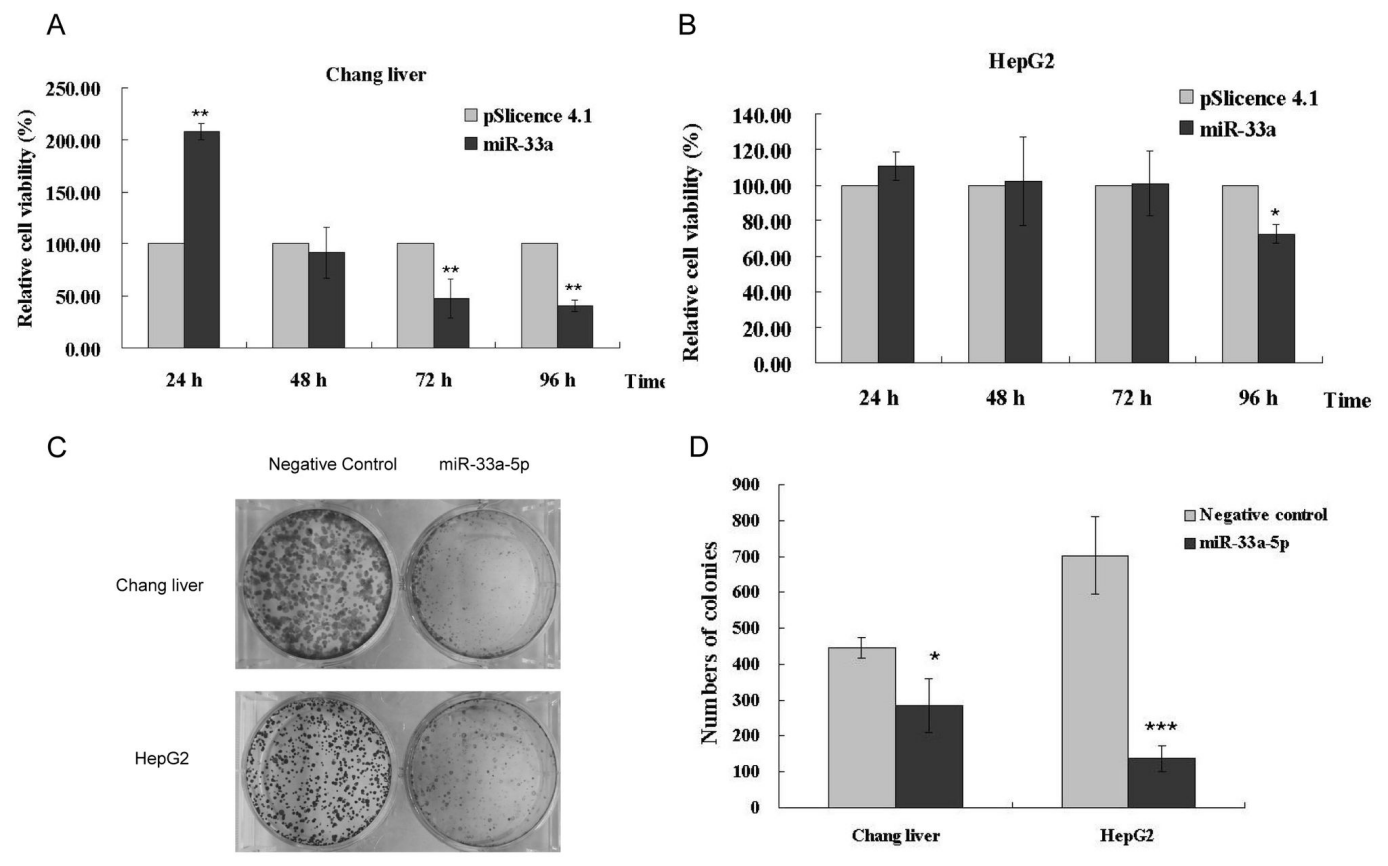
miR-33a inhibits HCC cell growth. The cell viability of Chang liver (A) and HepG2 (B) transfected with miR-33a expression vector is determined by MTT assay. (C) Colony formation of Chang liver and HepG2 transected with miR-33a-5p mimics. (D) Statistical analysis of colony numbers in (C). Data is presented as mean ± SD (n = 3). *P < 0.05, **P < 0.01, ***P <0.001.

Since β-catenin is an important component of the Wnt/β-catenin signaling pathway, negative regulation at the protein level by miR-33a must affect the Wnt/β-catenin signaling pathway and HCC cell growth as well. To illustrate this clearly, Chang liver and HepG2 cells were transfected with miR-33a expression vector. Cell proliferation rates of Chang liver and HepG2 transfected with the miR-33 over-expression construct were signiﬁcantly decreased in 96 h (Figure 5A and 5B), in comparison to cells transfected with empty vector. Additionally, compared with cells transfected with an empty vector, colony numbers of cells transfected with miR-33a expression construct decreased significantly (Figure 5C and 5D). All these results indicate that miR-33a can inhibit HCC cell proliferation*.*

### miR-33a directly regulates β-catenin negatively by binding to its 3’-UTR

**Figure 6:**
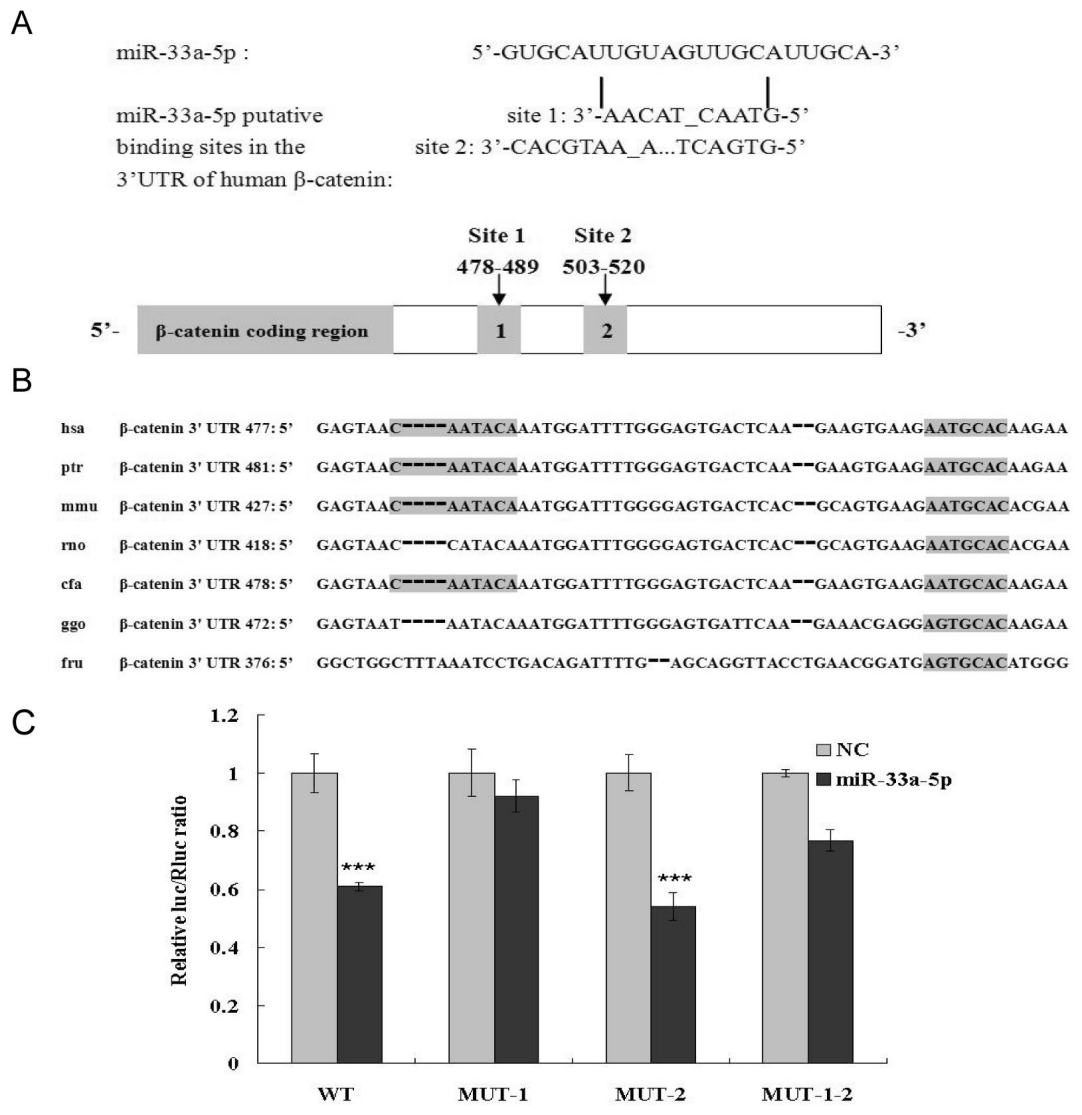
miR-33a directly and negatively regulates β-catenin by binding to its 3’-UTR. (A) Two putative binding sites of miR-33a-5p in the 3’-UTR of human β-catenin predicted by PicTar. The drawing is not to scale. (B) Target seed region (gray) of the miR-33a-5p binding sites were found within the 3’-UTR of β-catenin amongst different mammalian species. (C) miR-33a inhibites the luciferase activity of 3’-UTR of human β-catenin via binding site 1. The putative binding site is represented numerically in the figure. Wild-type and mutant human β-catenin 3’ UTR were independently constructed in the Luciferase Reporter System. They were co-transfected into 293T cells with pRL-TK vectors and miR-33a-5p mimics (50 nM). After 48 h of the transfection, luciferase activities were measured, and fireﬂy luciferase activities were normalized to Renilla luciferase activities. Data is presented as mean ± SD. *P < 0.05, **P < 0.01, ***P <0.001.

Based on the above evidence, we focused on whether miR-33a represses β-catenin by binding to the 3’-UTR of β-catenin mRNA directly. There are three *Homosapiens* transcript variants of β-catenin in the GenBank of the NCBI database, and their accessions in the GenBank are NM_001904 (transcript variant 1), NM_001098209 (transcript variant 2) and NM_001098210 (transcript variant 3). To ensure that all the putative binding sites of miRNA in the 3’-UTR of β-catenin were included, the 3’-UTR of β-catenin transcript variant 1 (the longest transcript variant of the three) was chosen as the full length 3’-UTR of β-catenin for the prediction of binding sites through bioinformatic analysis. Two putative miR-33 binding sites in the 3’-UTR of β-catenin were predicted by PicTar (Figure 6A). And they could be found in the 3’-UTR of all three human β-catenin transcript variants as they were highly conserved among different mammals (Figure 6B). The binding locations were 478~489 nt from the start of the 3’-UTR of human β-catenin (3091~3104 nt) and 503~520 nt from the start (3117~3141 nt).

To validate the putative miR-33a-5p binding sites in the 3’ UTR of β-catenin, a luciferase reporter system was employed. Wide-type and mutated 3’-UTR containing the putative binding sites were cloned into pMIR-luciferase vector. Cell 293T was co-transfected with miR-33a-5p mimics and the constructs. Figure 6C showed that the luciferase activity decreased greatly (about 2-fold) for the vector containing wild-type β-catenin 3’-UTR co-transfected with miR-33a-5p. Mutation of binding site 1 nearly recovered this decrease, whereas the mutation of binding site 2 retained the same luciferase activity as the wild type. These results demonstrated that the miR-33a-5p could directly regulate β-catenin negatively through its binding to the site 1 in the 3’ UTR of β-catenin.

### miR-33a-5p levels negatively correlate to levels of β-catenin in hepatoma carcinoma cells when exposed to AFB_1_

**Figure 7:**
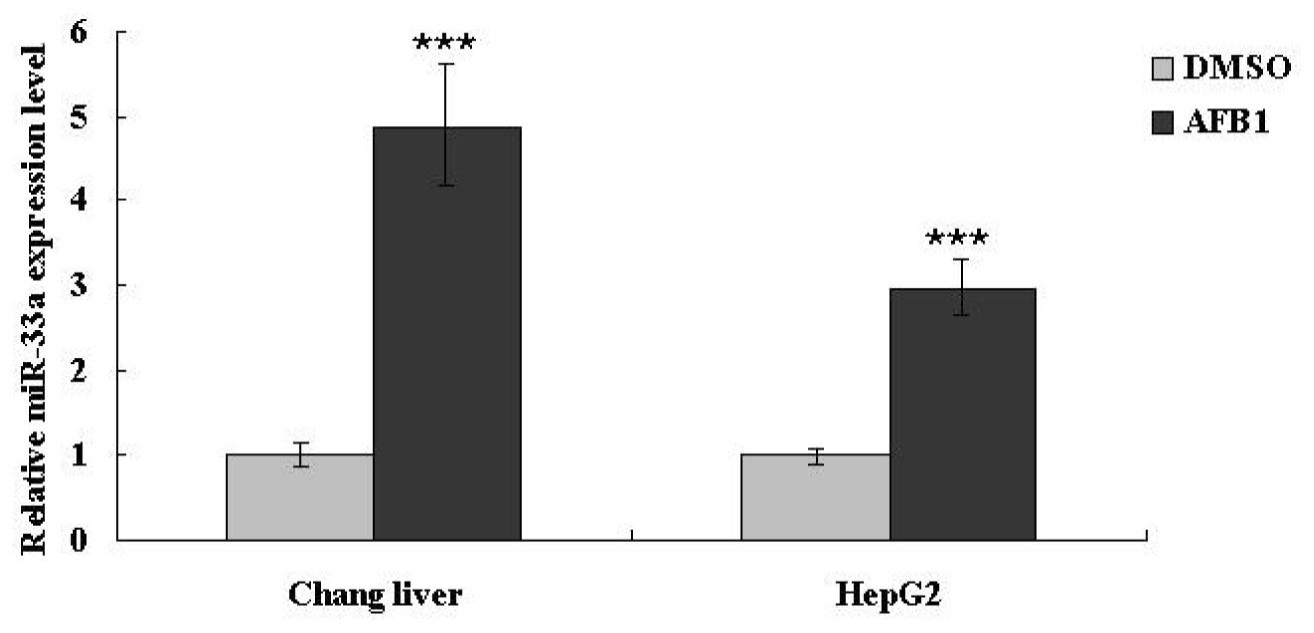
Expression of miR-33a-5p is up-regulated in hepatoma carcinoma cells after exposure to aﬂatoxin B_1_. Cells were exposed to AFB_1_ at their IC_50_ values, and expression of miR-33a-5p were anaylzed in 48 h. Same concentration of DMSO was included as control. Data is presented as mean ± SD. *P < 0.05, **P < 0.01, ***P <0.001.

We previously showed that AFB_1_ down-regulated β-catenin expression (Figure 1C and 1D), and β-catenin was thought to be the target of miR-33a. Thus, to further test whether AFB_1_ could induce abnormal expression of miR-33a in Chang liver and HepG2 cells, we treated both cell lines with AFB_1_ at their IC_50_ values, and performed qRT-PCR to detect miR-33a. The results indicated that the levels of miR-33a were up-regulated in both cell lines after AFB_1_ treatment (Figure 7). Together with the fact that AFB_1_ induced down-regulation of β-catenin (Figure 1C and 1D), these results revealed that the expression of miR-33a negatively correlated to the levels of β-catenin in cells which were exposed to AFB_1_.

### Model of AFB_1_ action: Down-regulation of β-catenin by up-regulation of miR-33a

**Figure 8:**
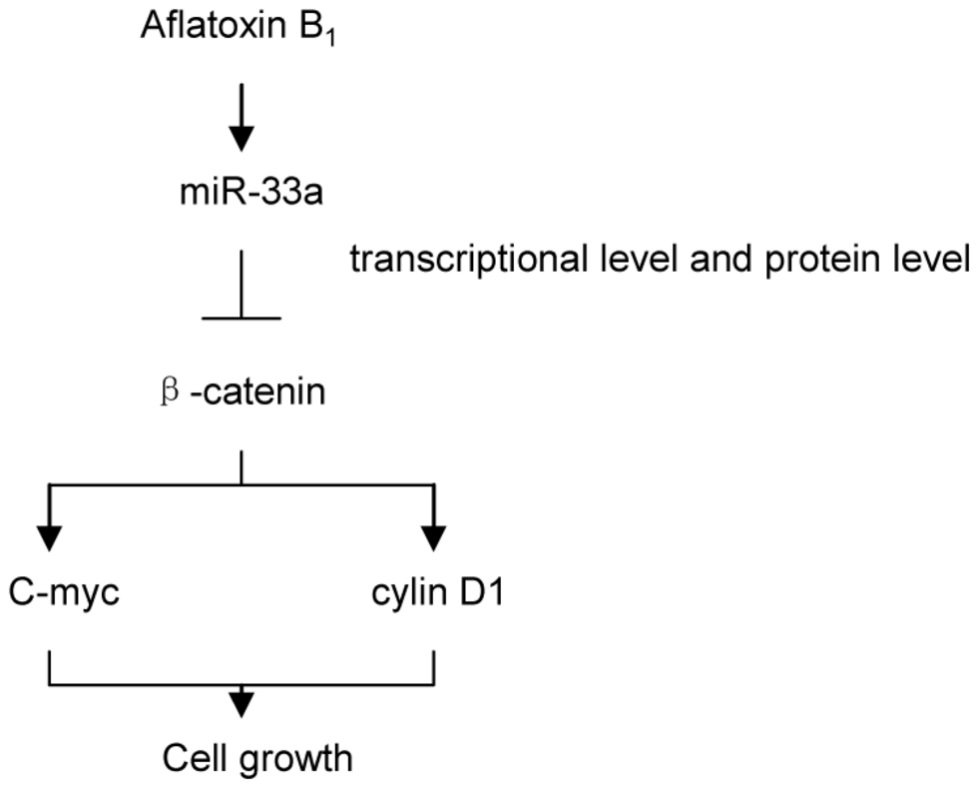
miRNAs negatively regulate β-catenin when cells are exposed to aﬂatoxin B_1_.

All these results showed that the expression of β-catenin decreased when the levels of miR-33a increased after AFB_1_ exposure. The negative correlation between miR-33a and β-catenin during exposure to AFB_1_ established the relationship between AFB_1_ and β-catenin mediated by miR-33a. As β-catenin is a necessary component in the Wnt/β-catenin signaling pathway, miR-33a appeared to be a bridge between AFB_1_ and the Wnt/β-catenin signaling pathway. Via the regulation of miR-33a, AFB_1_ negatively regulated the levels of β-catenin, which is classified as an oncogene. In fact, along with the decrease in expression of β-catenin, two genes downstream of β-catenin in Wnt/β-catenin signaling pathway, cyclin D1 and C-myc (two oncogenes), were also down-regulated in cells exposed to AFB_1_ (Figure 3C and 3D). Following these changes in Wnt/β-catenin signaling pathway, cell growth was repressed. Our results revealed a novel regulatory mechanism of β-catenin in cells exposed to AFB_1_ via miRNAs (Figure 8).

## Discussion

AFB_1_ contaminates cereals around the globe, and is regarded as one of the most dangerous carcinogens known to date. It appears to have an acute and chronic toxicity in every organ, in animals and humans. An exposure to AFB_1_, compounded with alcoholism or infection with HBV/ HCV, would most easily cause the suppression of immunity and increase susceptibility to diseases such as the development of liver disease and HCC [42]. Previously, reports indicated that AFB_1_ induced cell cytotoxicity or apoptosis in a dose- and time-dependent manner [43,44]. The cytotoxic effect of AFB_1_ was not always associated to apoptosis, and it could be induced by substantial genotoxicity and the decreased defense ability [45].

Exposure to AFB_1_ via food or even traditional Chinese medicine has been suggested to increase the risk of HCC [46]. Therefore searching for potential biomarkers to detect early hepatotoxicity induced by AFB_1_ or a novel molecular therapy for hepatoma caused by AFB_1_ becomes necessary. Till now, most of the molecular mechanisms known about the pathogenicity and carcinogenicity of AFB_1_ were carried out at a protein level, and very little was observed on the basis of miRNAs. Our research is the first study of AFB_1_ based on miRNA, and hence, it offers the possibility to find some miRNAs for the detection of early acute hepatotoxicity induced by AFB_1_ and provides the new tools for diagnosis, prognosis and therapy in HCC.

The tracks of miRNAs are found in almost all biological processes, from normal physiological functions of cells to the etiology of diseases. It was been reported that miR-33 could inhibit cell apoptosis and control hematopoietic stem cells (HSC) self-renewal by targeting *p53* [47,48], and that this function of miR-33 could be applied to the prevention and treatment of hematopoietic disease. Recently, miR-33 was shown to regulate cell proliferation and cell cycle by inhibiting the expression of the cyclin-dependent kinase 6 (CDK6) and cyclin D1 (CCND1). miR-33a, belonging to the miR-33 family, regulates receptor-interacting protein 140 (RIP140) in inflammatory cytokine production, by reducing RIP140 coactivator activity for NF-κB, and hence decreasing NF-κB reporter activity and thus the inflammatory potential in macrophages [49]. In this study, AFB_1_ exposure caused the up-regulation of miR-33a and reduction of β-catenin. miR-33a was demonstrated to negatively regulate β-catenin on both post-transcriptional and protein levels. In fact, miR-340 was also observed to be involved in the down-regulation of β-catenin in our research, so we believe that miR-33 and miR-340 may play their functions in a synergistic manner on the pathogenicity and carcinogenicity related to AFB_1_. Although we didn’t explore the function of miR-340 here, it is still worthy of investigation in the future. With this study as a basis, more miRNAs involved with AFB_1_ might be identified in the future.

We know that β-catenin is a critical component of the well-studied Wnt/β-catenin signaling pathway. By down-regulating of β-catenin, miR-33a negatively regulated the downstream genes (C-myc and cyclin D1) of β-catenin in Wnt/β-catenin pathway, and also inhibited cells growth upon exposure to AFB_1_. Considering that GSK-3β generally mediates phosphorylation and subsequent degradation of β-catenin, the expression level of GSK-3β was examined to exclude the possibility that it might be involved in the down-regulation of β-catenin after miR-33a overexpression. If the down-regulation of β-catenin was caused via GSK-3β, the expression of GSK-3β must be up-regulated. However, as shown in Figure 3B, the expression of GSK-3β was not up-regulated. This leads to the conclusion that GSK-3β does not take part in the down-regulation of β-catenin mediated by miR-33a. In Figure 3B and 3C, the mRNA level of β-catenin does not shown a significant difference in Chang liver cells while the reduction of C-myc mRNA level appeared to be significant. We conjectured that miRNA had a distinct effect on target mRNA that is dependent on the context which is consistent with the research of Hu [50]. While we detected the mRNA levels of β-catenin and C-myc in Chang liver cells, the degree of β-catenin translation repression may be more than its mRNA degradation, or maybe the translation repression of β-catenin by miR-33a could have occurred before β-catenin mRNA degradation. Alternatively, perhaps C-myc reduction is caused by miR-33a directly or by the other pathways affected by miR-33a. Moreover, mRNA destruction could be affected by many factors, like the preference pathway for mRNA decay, the miRNA ribonucleoprotein complex (miRNP), decay factors and so on [50]. These factors would have positive or negative effects on stability, translation, transport, localization, and polyadenylation/ deadenylation which affect the level of mRNA destruction [50–53].

In summary, we preliminarily established the relationship between miR-33a and AFB_1_ in liver cells. AFB_1_ negatively regulated β-catenin by overexpression of miR-33a, and then participated in the regulation of the β-catenin signaling pathway and cell growth. Our results revealed a novel regulatory effect of AFB_1_ on β-catenin and Wnt/β-catenin pathway. It opened a new view of the pathogenicity and carcinogenicity of AFB_1_ via the Wnt/β-catenin pathway. It also disclosed a novel mode of AFB_1_ toxicity to cells mediated by miRNA and shed light on the function of miR-33a in the regulation of cell proliferation and cancer generation. This might open up some new possibilities for future therapeutic intervention.

## Materials and Methods

### Cell lines, RNA oligoribonucleotides and vectors construction

Chang liver, HepG2, Bel-7404, AGS and HeLa cell lines were purchased from a typical cell culture collection Committee of the Chinese Academy of Sciences Library, and cultured in RPMI medium 1640 supplemented with 10% FBS (fetal bovine serum, Biotechnology Co. Ltd., Shanghai, China). The sequences of miRNA mimics (from RiboBio Co. Ltd., Guangzhou, China) are listed in the Table S2. Primers (Biotechnology Co. Ltd., Shanghai, China) used during the construction of miRNA expression vectors are listed in the Table S3.

miRNA precursors and their 5’- and 3’- ﬂanking sequences were amplified and cloned into the *Bam*HI and *Hin*d Ⅲ sites in the pSilencer 4.1-CMV expression vector. The wild-type 3’-UTR of β-catenin was amplified and cloned into the *Bam*HI and *Hin*d Ⅲ sites downstream of the ﬁreﬂy luciferase reporter gene in the ﬁreﬂy pMIR-luciferase reporter vector. The mutated 3’-UTR of β-catenin (sequences listed in Table S4) were synthesized with *Bam*HI and *Hin*d Ⅲ sites, and then cloned into the ﬁreﬂy pMIR-luciferase reporter vector too. Sequences were confirmed by DNA sequencing. Transfections were done in triplicates for each independent experiment by Lipofectamine 2000 (Life Technologies Corporation, Shanghai, China) according to the manufacturer’s instructions.

### IC_50_ determinations

5 mg of AFB_1_ (Sigma-aldrich, Saint Louis, USA) were dissolved into 500 μL of DMSO. Cells were plated into 96-wells plates, and then exposed to AFB_1_ at different concentrations (4, 8, 16, 32, and 64 μg/mL). Since DMSO was required for the solublization of AFB_1_, the same concentrations of DMSO were used in the controls for this assay. Cell viabilities were determined at 550 nm by way of MTT cell proliferation and cytotoxicity detection kit (KeyGEN Biotech, Nanjing, China) according to the manufacturer’s instructions after 48 h, and then the relative cell viability of cells were calculated. The concentration of AFB_1_ that inhibited 50% of the cell viability was extrapolated from relative cell viability vs. [log concentration] curves, and then used to obtain IC_50_ values.

### Western blot

After AFB_1_ exposure or miRNA overexpression for 48 h, cells were lysed and separated in 7.5% SDS-PAGE, and then transferred to PVDF membranes (BioTrace^TM^, Utah, USA) by semi-dry electrotransfer. The PVDF membranes were blocked by 5% fat-free milk powder dissolved in TBS buffer containing 0.1% Tween-20. Antibody against β-catenin (Santa Cruz Biotechnology, CA, USA) and BeyoECL Plus (Beyotime Institute of Biotechnology, Shanghai, China) were used for detection. Band intensity of western blots was analyzed with Scion Image software (Scion Corporation, MD, USA) and normalized to β-actin or GADPH (Beyotime Institute of Biotechnology, Shanghai, China).

### Real time PCR

The total RNA of cells was extracted with TRIpure (BioTeke corporation, Beijing, China) as suggested by the manufacturer after miRNA mimics transfection or AFB_1_ exposure for 48 h. cDNA synthesis was carried by a First Strand cDNA Synthesis Kit (Fermentas, St Leon-Rot, Germany). Primers for the real time PCR were listed in Table S5. The RT-primer of miR-33a was designed according to Chen et al. [54]. Real time PCR was performed by SYBR^®^ *Premix Ex Taq* ^TM^ (Takara, Dalian, China) according to the manufacturer’s instructions in Mastercycler® ep realplex real-time PCR system (Eppendorf, Hamburg, Germany).

### Cell viability and cell colony formation assay

Cell viability was determined by the MTT Cell Proliferation and Cytotoxicity Detection Kit (KeyGEN Biotech, Nanjing, China) following the manufacturer’s instructions. In brief, cells were transfected in a 96-well plate for 4 d, and the cell viability was determined at 550 nm each day. For colony formation assay, cells were digested by trypsin (Solarbio Science & Technology Co., Ltd, Beijing, China) after transfection for 48 h, and 500 cells per well were replated on a fresh 6-well plate. Cells were maintained in RPMI medium 1640 supplemented with 10% FBS, penicillin and streptomycin for 2 weeks. The cells were then fixed with methanol and stained with 0.1% crystal violet and the colony numbers per well were counted.

### Luciferase assay

Luciferase assay was performed by RiboBio Co., Ltd (Guangzhou, China). Details were presented below. miR-33a mimics (50 nM) or negative control were cotransfected with pMIR-luciferase reporter vectors (100 ng/mL, wild-types or mutants) and pRL-TK vectors (5 ng/mL, as a normalization for transfection efficiency) into 293T cells in 96-well plates by using Lipofectamine 2000. Dual-Luciferase® Reporter Assay System (Promega Corporation, Beijing, China) was employed to measure the luciferase activities at 24 h after transfection. All measurements were performed in triplicates and repeated in independent experiments at least thrice.

### Statistics analysis

Data were presented as mean ± SD. The statistical significance of differences was assessed using one way ANOVA in Microsoft Excel. P < 0.05 meant that the differences were statistically significant. 

## Supporting Information

Table S1 The miRNA candidates for targeting β-catenin. The table contains 5 miRNA candidates predicted by three onlines computational algorithms(PicTar, miRNA Viewer and Targetscan). They are ranked based on the number of binding sites found in the 3’-UTR of human β-catenin and the frequencies of prediction by three computational algorithms.
(DOC)

Table S2 Sequences of miRNA mimics. miR-33a-5p and miR-33a-3p are synthezied according to miRBase (http://www.mirbase.org/). Two miRNA mimics and their miRNA mimics negative control are purchased from RiboBio Co. Ltd., Guangzhou, China.
(DOC)

Table S3 Primers for miRNA expression vector construction. Primers are designed by Primer Premier 5.0 software and synthezied by Biotechnology Co. Ltd., Shanghai, China.
(DOC)

Table S4 Sequence for 3’-UTR construction of β-catenin. Primers of the wild-type 3’-UTR of β-catenin are designed by Primer Premier 5.0 software and used to amplified the wild-type 3’-UTR of β-catenin. The primers and three mutated 3’-UTR of β-catenin sequences are synthezied by Biotechnology Co. Ltd., Shanghai, China.
(DOC)

Table S5 Primers for real time PCR. Primers of U6 and miR-33a-5p are designed by Primer Premier 5.0 software and other primers indicated with * are gotten from RTPrimerDB. All primers are synthezied by Biotechnology Co. Ltd., Shanghai, China.
(DOC)
