# Supplementary material for: Aflatoxin B1 Negatively Regulates Wnt/β-Catenin Signaling Pathway through Activating miR-33a
Source: PLoS One. 2013 Aug 27;8(8):e73004. doi: 10.1371/journal.pone.0073004 (PMC3754916; doi:10.1371/journal.pone.0073004)
Supplement: Table S1 — The table contains 5 miRNA candidates predicted by three onlines computational algorithms(PicTar, miRNA Viewer and Targetscan). They are ranked based on the number of binding sites found in the 3’-UTR of human β-catenin and the frequencies of prediction by three computational algorithms. (DOC) [file pone.0073004.s001.doc]

**Table S1 The miRNA candidates for targeting β-catenin.**

| **miRNA** | **Total potential**  **binding sties** | **Numbers of**  **Computational algorithm** |
| --- | --- | --- |
| miR-320a | 5 | 1 |
| miR-33a | 2 | 1 |
| miR-139 | 2 | 1 |
| miR-214 | 2 | 2 |
| miR-125a | 1 | 2 |
| miR-340 | 1 | 2 |
