# Supplementary material for: Aflatoxin B1 Negatively Regulates Wnt/β-Catenin Signaling Pathway through Activating miR-33a
Source: PLoS One. 2013 Aug 27;8(8):e73004. doi: 10.1371/journal.pone.0073004 (PMC3754916; doi:10.1371/journal.pone.0073004)
Supplement: Table S2 — miR-33a-5p and miR-33a-3p are synthezied according to miRBase (http://www.mirbase.org/). Two miRNA mimics and their miRNA mimics negative control are purchased from RiboBio Co. Ltd., Guangzhou, China. (DOC) [file pone.0073004.s002.doc]

**Table S2** Sequences of miRNA mimics.

| **hsa-miRNA** | **Sequence (5’→3’)** |
| --- | --- |
| miR-33a-5p | GUGCAUUGUAGUUGCAUUGCA |
| miR-33a-3p | CAAUGUUUCCACAGUGCAUCAC |
| miRNA mimics negative control | UUUGUACUACACAAAAGUACUG |
