# Supplementary material for: Aflatoxin B1 Negatively Regulates Wnt/β-Catenin Signaling Pathway through Activating miR-33a
Source: PLoS One. 2013 Aug 27;8(8):e73004. doi: 10.1371/journal.pone.0073004 (PMC3754916; doi:10.1371/journal.pone.0073004)
Supplement: Table S3 — Primers are designed by Primer Premier 5.0 software and synthezied by Biotechnology Co. Ltd., Shanghai, China. (DOC) [file pone.0073004.s003.doc]

**Table S3 Primers for miRNA expression vector construction.**

| **miRNA** | **Action of primer** | **Sequence (5’→3’)** |
| --- | --- | --- |
| miR-33a | Forward primer | GGTTAGATCTTGCTCCAGCGGTTTG |
|  | Reverse primer | GTAAAGCTTGCCCTCCTGTTTCCTG |
| miR-125 | Forward primer | CCTCCTTCCCCTGAAATCTGT |
|  | Reverse primer | GGTCTCCTCACTGATTTCGGT |
| miR-139 | Forward primer | CCTGCTCTTTCCCTCTTCCCATTCC |
|  | Reverse primer | GCCAAAGCTTACCTGCCAGAGACCT |
| miR-214 | Forward primer | ACACGGATCCAACAGGCTGATTGTATCT |
|  | Reverse primer | TGGCGAAGCTTTAAAGGTCAAGGGTAGT |
| miR-320a | Forward primer | ACACGGATCCCAGCCGCCAGCCTTCGGTCTCC |
|  | Reverse primer | CCCGGAAGCTTCGCCGCAGCATAGCGTAGCCC |
| miR-340 | Forward primer | GGGGAAGATCTCTTTGAATAGAATCACATGG |
|  | Reverse primer | GCCCAAGCTTGTTGTGATCAGTAAATTAGAG |
