# Supplementary material for: Aflatoxin B1 Negatively Regulates Wnt/β-Catenin Signaling Pathway through Activating miR-33a
Source: PLoS One. 2013 Aug 27;8(8):e73004. doi: 10.1371/journal.pone.0073004 (PMC3754916; doi:10.1371/journal.pone.0073004)
Supplement: Table S4 — Primers of the wild-type 3’-UTR of β-catenin are designed by Primer Premier 5.0 software and used to amplified the wild-type 3’-UTR of β-catenin. The primers and three mutated 3’-UTR of β-catenin sequences are synthezied by Biotechnology Co. Ltd., Shanghai, China. (DOC) [file pone.0073004.s004.doc]

**Table S4 Sequence for 3’-UTR construction of β-catenin.**

| **Gene** | **primer** | **Sequence (5’→3’)** |
| --- | --- | --- |
| β-catenin-3'UTR | Forward | CGGGGGCACTAGTATCATCCTTTAGGTAAG |
|  | Reverse | CGGGGCAAGCTTAGTTTAATTCTGAACC |
| MUT-1 |  | CTAGACTAGTTTGAAGTAAACTTTTTGTTCTGGTCCTTTTTGGTCGAGGAGTAtCAAatgtAATGGATTTTGGGAGTGACTCAAGAAGTGAAGAATGCACAAGAATGGATCACAAGATGGAATTTATCAAACCCTAGCCTTaagcttCC |
| MUT-2 |  | CTAGACTAGTTTGAAGTAAACTTTTTGTTCTGGTCCTTTTTGGTCGAGGAGTAACAATACAAATGGATTTTGGGAGTGtCaCAAGAAGTGAAGAtTcgtgAAGAATGGATCACAAGATGGAATTTATCAAACCCTAGCCTTaagcttCC |
| MUT-1-2 |  | CTAGACTAGTTTGAAGTAAACTTTTTGTTCTGGTCCTTTTTGGTCGAGGAGTAtCAAatgtAATGGATTTTGGGAGTGtCaCAAGAAGTGAAGAtTcgtgAAGAATGGATCACAAGATGGAATTTATCAAACCCTAGCCTTaagcttCC |
