# Supplementary material for: Aflatoxin B1 Negatively Regulates Wnt/β-Catenin Signaling Pathway through Activating miR-33a
Source: PLoS One. 2013 Aug 27;8(8):e73004. doi: 10.1371/journal.pone.0073004 (PMC3754916; doi:10.1371/journal.pone.0073004)
Supplement: Table S5 — Primers of U6 and miR-33a-5p are designed by Primer Premier 5.0 software and other primers indicated with * are gotten from RTPrimerDB. All primers are synthezied by Biotechnology Co. Ltd., Shanghai, China. (DOC) [file pone.0073004.s005.doc]

**Table S5** Primers for real time PCR.

| **Gene** | **Primer** | **Sequence (5’→3’)** |
| --- | --- | --- |
| U6 | Forward primer | CGGTCGCTTCGGCAGCACAT |
|  | Reverse primer | GGCGGTTTGCGTGTCATCCTTG |
| miR-33a-5p | RT-primer | GTCGTATCCAGTGCAGGGTCCGAGGTATTCGCACTGGATACGACTGCAATGC |
|  | Forward primer | GGTGCATTGTAGTTGCATTGC |
|  | Reverse primer | GTGCAGGGTCCGAGGTATTC |
| β2-MG* | Forward primer | CTCCGTGGCCTTAGCTGTG |
|  | Reverse primer | TTTGGAGTACGCTGGATAGCCT |
| β-catenin* | Forward primer | GCTGGGACCTTGCATAACCTT |
|  | Reverse primer | ATTTTCACCAGGGCAGGAATG |
| GSK-3β* | Forward primer | CTCCTCATGCTCGGATTCA |
|  | Reverse primer | TGCAGAAGCAGCATTATTGG |
| C-myc* | Forward primer | GCCACGTCTCCACACATCAG |
|  | Reverse primer | TGGTGCATTTTCGGTTGTTG |
| cyclin-D1* | Forward primer | CGCCCCACCCCTCCAG |
|  | Reverse primer | CCGCCCAGACCCTCAGACT |

* indicated the primers were from RTPrimerDB
